# Perceptions of activity-supportive environment and motorcycle use among urban Taiwanese adults

**DOI:** 10.1186/s12889-017-4682-0

**Published:** 2017-08-18

**Authors:** Chien-Yu Lin, Yung Liao

**Affiliations:** 1 Institute of Health Behaviors and Community Sciences, National Taiwan University, 17, Xuzhou Road, Taipei, 100 Taiwan; 2 Department of Health Promotion and Health Education, National Taiwan Normal University, 162, Heping East Road Section 1, Taipei, 106 Taiwan

**Keywords:** Motorcycle use, Perception of environment, Urban adults, Sedentary behavior, Transportation, Supportive environments

## Abstract

**Background:**

Although research has shown that numerous perceived environmental factors are supportive of physical activity, little is known about their associations with sedentary transport in motorcycle-oriented countries. This study examined the association between perceptions of Taiwan’s environmental factors and urban adults’ motorcycle use.

**Methods:**

Cross-sectional data from 1003 Taiwanese adults aged 20–64 years from three urban cities were collected through telephonic surveys in 2015. Data on motorcycle use, sociodemographic variables, and perceived environmental attributes were obtained. Logistic regression analyses were performed.

**Results:**

In Model 1, adults who perceived favorable access to public transport and destinations, presence of sidewalks, and safety from crimes at night were less likely to use motorcycles. In Model 2, in which potential covariates were additionally adjusted for, the same four environmental attributes (perceived favorable access to public transport and destinations, presence of sidewalks, and safety from crimes at night; odds ratio [OR] = 0.46, 0.65, 0.63, 0.64, respectively) were significantly associated with motorcycle use.

**Conclusion:**

The investigated perceived environmental factors, which have previously been associated with facilitating active transportation, discourage sedentary modes of transport, such as motorized vehicles.

## Background

Excessive sitting is associated with increased risks of cardiometabolic diseases, Type 2 diabetes, specific types of cancer, and premature mortality [1]. Sitting, especially while commuting or traveling, is a common sedentary behavior known to adversely affect human health [2, 3]. More than 70% of adults in Taiwan use private motorized vehicles as their main mode of daily travel [4], which is comparable with the rates in Australia and the United States [5, 6]. In addition, Taiwan has a considerably high motorcycle ownership rate (608.5 per 1000 people) [7], and nearly half of Taiwan’s adult population (49.6%) use motorcycles as their main transportation mode to travel to work and other destinations. The health risks associated with sedentary transport [8–10] renders critical the development of effective strategies to reduce motorcycle use through initiatives in urban planning and design.

To be effective, according to ecological models of health behavior, such urban planning and design initiatives should be based on a comprehensive understanding of local perceptions of neighborhood environmental factors associated with motorcycle use [3, 11, 12]. Research from car-oriented countries have not adequately examined walkability- and driving-related problems [13–15]. Moreover, the mechanism through which perceived neighborhood environmental factors effectively induce motorcycle use among urban Taiwanese adults remains unclear. The residents’ experience of their neighborhood environment is critical to understanding their transportation choices. However, several specific perceptions of the neighborhood environment, such as the sense of safety or aesthetics, are difficult to measure objectively [16]. Furthermore, although numerous studies have examined the associations between perceptions of the neighborhood environment with overall sitting time [17, 18], domain-specific sedentary behavior such as television viewing [19], and transport-related sitting [17], fewer studies have focused on specific forms of transport-related sedentary behavior, such as motorcycle use, in Asia. Research from motorcycle-oriented countries can strengthen evidence for the associations of perceived neighborhood environmental factors with motorcycle use. These perceptions have been found to be related to higher levels of walking and cycling [20–22], and conversely, tended to engage lower levels of motorcycle use. In this study, therefore, we examined perceived neighborhood environmental factors associated with motorcycle use among Taiwan’s urban adults.

## Methods

### Participants

Cross-sectional survey data used in this study were obtained through a random-digit dialing telephone-based survey conducted over a period of one month from September to October 2015 by a telephone research company in three Taiwanese cities: Taipei City, the capital of Taiwan (area: 271.7 km^2^; population: 2,702,315); New Taipei City, the largest city in Taiwan (area: 2052.6 km^2^; population: 3,966,818); and Kaohsiung City, the second largest city in Taiwan (area: 2947.6 km^2^; population: 2,778,992). Those three cities have comprehensive transport infrastructure, including public bicycle system, public transportation, and pedestrian sidewalks. Respondents were selected through a stratified random sampling process. The eligible respondents were Mandarin-speaking adults aged 20–64 years with private household telephones. We categorized the respondents according to their cities of residence as Taipei City, New Taipei City, and Kaohsiung City. Furthermore, the respondents were stratified by age (20–29, 30–39, 40–49, 50–59, and 60–64 years) and gender. For each city, the telephone numbers were selected at random by a computer-assisted system. The interviewers, who received two days of training before the survey period, used a standardized questionnaire and were experienced in administering population-based telephonic surveys. For ensuring data reliability, the length of each interview was restricted to 20 min to maintain the respondent’s interest [23]. Of the 5333 adults contacted, 1069 completed the survey (response rate: 20.04%). After data cleaning, 1003 valid responses were obtained. Respondents were offered no rewards for participation in the survey. According to Research Ethics Committee (REC) of National Taiwan Normal University, telephone survey targeting adults has been considered to be minimal-risk for respondents and qualify for a verbal consent process. According to the approved procedure and template, verbal consent was obtained and recorded before the start of each interview. The study protocols were reviewed and approved by the REC of National Taiwan Normal University (REC number: 201504HM005).

### Outcome variable

The outcome variable was self-reported motorcycle use (riders and passengers were both included); data for this variable were acquired through the question “How many times have you used a motorcycle in the past 7 days?” The frequency distribution was skewed; hence, the variable of motorcycle use was dichotomized as indicators of use (yes) and nonuse (no) in the previous 7 days.

### Perceptions of the neighborhood environment

The variables comprising perceived neighborhood environmental factors were determined and measured using the Taiwanese version of the International Physical Activity Questionnaire Environmental Module (IPAQ-E). The IPAQ-E was developed as part of the International Physical Activity Prevalence Study to investigate the relationship between perceptions of the environment and walking or cycling in several countries [24–26]. The Taiwanese version of the IPAQ-E was used in a previous study on the association between perceptions of the environment and active transportation [22]. The IPAQ-E questionnaire comprises three categories of items, with seven core items, four recommended items, and six optional items [27]. Of these 17 items, our study utilized the following seven, based on previous studies [12, 28]; the definitions of the items are provided within parentheses: (1) residential density (main type of housing in the neighborhood); (2) access to public transportation (10–15 min to a transit stop); (3) presence of sidewalks (presence of sidewalks on most streets); (4) safety from crime at night (crime rate, which may render night-time walks unsafe); (5) aesthetic experiences (the presence of interesting elements that make walking outside attractive); (6) street connectivity (the presence of numerous four-way intersections); and (7) destinations (the presence of numerous places of interest within easy walking distance). One personal item (vehicle ownership), three core items (access to shops, presence of bicycle lanes, access to recreational facilities), two recommended items (traffic safety, visibly active people), and four optional items (sidewalk maintenance, bicycle-lane maintenance, safety from crime during day time, and the safety of cyclists in traffic) were not included as environmental variables. Each question was answered on a 4-point Likert scale ranging from “strongly disagree” to “strongly agree.” An additional option, *unknown/uncertain*, was also provided; responses with this option were treated as missing data in the analysis. Following previous studies [22, 25], the seven perceived environmental factors were transformed into binary items. Residential density was dichotomized as “detached single-family housing” and “other” (e.g., townhouses, row houses, apartments, and condominiums); the other eight items were dichotomized as “agree” (“strongly agree” and “somewhat agree”) and “disagree” (“somewhat disagree” and “strongly disagree”).

### Sociodemographic variables

The sociodemographic variables recorded in the survey were gender, age, city of residence, education level, occupation type, marital status, body mass index (BMI), and vehicle ownership (which included household motorcycle and car ownership). These variables were further categorized as follows: Age was divided into five categories: a) 20–29; b) 30–39; c) 40–49; d) 50–59; and e) 60–64 years. Education level was categorized into a) high school degree or lower (including elementary school, junior high school, high school, and vocational school degree) and b) university degree or higher. Occupation type was divided into two categories: a) part-time and b) full-time. Marital status was divided into a) married and b) unmarried (including widowed, separated, and divorced). BMI was calculated from the self-reported weight and height and was categorized as a) not overweight (<24 kg/m^2^), or b) overweight or obese (≥24 kg/m^2^), according to the threshold for Taiwan [29]. In addition, the number of vehicles owned was recorded; accordingly, household motorcycle and car ownership was dichotomized as “yes” (if >0) and “no” (if 0).

### Statistical analyses

We entered each of perceived environmental factors into logistic regression with adjusted to examine the associations of the seven environmental factors with motorcycle use. Two statistical models were constructed for the analyses. Model 1 was an unadjusted model. The covariates of the sociodemographic variables of gender, age, education level, occupation, marital status, and BMI were adjusted for in Model 2. The odds ratio (ORs) and 95% confidence interval (CIs) were calculated for each variable. Inferential statistical analysis was performed using IBM SPSS 22.0 software (IBM, Armonk, NY, USA), and the level of significance was set at *p* < 0.05.

## Results

### Participant characteristics

Table 1 presents the respondent characteristics (mean age: 44.5 ± 12.2 years). The distribution of the three covariates of gender, age, and city of residence in this sample were balanced. Among all respondents, 62.9% had a university degree or a higher level of education, 69.3% had a full-time job, 66.9% were married, 40.2% were overweight or obese, and 87.4% and 79.8% owned one or more motorcycles and cars, respectively. In addition, 63.1% of the respondents had used a motorcycle in the 7 days prior to the survey.

**Table 1:** Basic characteristics of the respondents (*N* = 1003)

Basic Characteristics	Total	Motorcycle Use		*p*-Value
		Yes	No	
N (%)	1003	633 (63.1%)	370 (36.9%)	
Gender				<0.001^**^
Men	497 (49.6%)	342 (54.0%)	155 (41.9%)	
Women	506 (50.4%)	291 (46.0%)	215 (58.1%)	
Age (year)				0.027^*^
20–29	138 (13.8%)	86 (13.6%)	52 (14.1%)	
30–39	227 (22.6%)	153 (24.2%)	74 (20.0%)	
40–49	249 (24.8%)	171 (27.0%)	78 (21.1%)	
50–59	257 (25.6%)	145 (22.9%)	112 (30.3%)	
60–64	132 (13.2%)	78 (12.3%)	54 (14.6%)	
Residential city				<0.001^**^
Taipei city	334 (33.3%)	155 (24.5%)	179 (48.4%)	
New Taipei city	342 (34.1%)	211 (33.3%)	131 (35.4%)	
Kaohsiung city	327 (32.6%)	267 (42.2%)	60 (16.2%)	
Educational level				0.009^*^
High school degree and lower	372 (37.1%)	254 (40.1%)	118 (31.9%)	
University and higher	631 (62.9%)	379 (59.9%)	252 (68.1%)	
Occupational type				0.058
Not full-time	308 (30.7%)	181 (28.6%)	127 (34.3%)	
Full-time	695 (69.3%)	452 (71.4%)	243 (65.7%)	
Marital status				0.145
Not married	332 (33.1%)	220 (34.8%)	112 (30.3%)	
Married	671 (66.9%)	413 (65.2%)	258 (69.7%)	
Body Mass Index				0.013^*^
Non-overweight	600 (59.8%)	360 (56.9%)	240 (64.9%)	
Overweight/obese	403 (40.2%)	273 (43.1%)	130 (35.1%)	
Household motorcycle ownership				<0.001^**^
Yes	877 (87.4%)	631 (99.7%)	246 (66.5%)	
No	126 (12.6%)	2 (0.3%)	124 (33.5%)	
Car ownership				0.759
Yes	800 (79.8%)	503 (79.5%)	297 (80.3%)	
No	203 (20.2%)	130 (20.5%)	73 (19.7%)	

^*^ *p* < 0.05; ^**^ *p* < 0.001

### Perceived neighborhood environmental factors associated with motorcycle use seven days prior to the survey

Logistic regression analysis of Model 1, which was unadjusted, demonstrated that four of the seven attributes of perceptions of the environment were significantly associated with motorcycle use (Table 2). Respondents who perceived favorable access to public transport (OR = 0.45; 95% CI: 0.25–0.82), the presence of sidewalks (OR = 0.67; 95% CI: 0.48–0.90), safety from crimes at night (OR = 0.64; 95% CI: 0.46–0.89), and convenient access to destinations (OR = 0.66; 95% CI: 0.47–0.95) were less likely to have used motorcycles in the past 7 days. After adjustment for potential confounders in Model 2, the same four attributes were significantly associated with motorcycle use. Respondents who perceived favorable access to public transport (OR = 0.46; 95% CI: 0.26–0.84), the presence of sidewalks (OR = 0.63; 95% CI: 0.46–0.87), safety from crimes at night (OR = 0.64; 95% CI: 0.45–0.89), and good access to destinations (OR = 0.65; 95% CI: 0.45–0.94) were associated with lower levels of motorcycle use in the 7 days prior to the survey.

**Table 2:** Perceived environmental factors associated with motorcycle use among Taiwanese adults

Perceived Environmental Factors			Motorcycle Use	
			Model 1	Model 2
	N	%	OR (95% CI)	OR (95% CI)
Residential density				
High	955	95.2%	0.69(0.37–1.31)	0.62(0.32–1.20)
Low	48	4.8%	1.00	1.00
Access to public transport				
Good	934	93.1%	0.45(0.25–0.82)^**^	0.46(0.26–0.84)^*^
Poor	69	6.9%	1.00	1.00
Presence of sidewalks				
Yes	767	76.5%	0.67(0.48–0.90)^**^	0.63(0.46–0.87)^**^
No	236	23.5%	1.00	1.00
Safety from crimes at night				
Safe	207	20.6%	0.64(0.46–0.89)^**^	0.64(0.45–0.89)^**^
Not safe	796	79.4%	1.00	1.00
Aesthetics				
Yes	601	59.9%	0.94(0.73–1.23)	0.99(0.75–1.29)
No	402	40.1%	1.00	1.00
Connectivity of streets				
Yes	772	77.0%	1.02(0.75–1.38)	1.05(0.77–1.44)
No	231	23.0%	1.00	1.00
Presence of destination				
Yes	829	82.7%	0.66(0.47–0.95)^*^	0.65(0.45–0.94)^*^
No	174	17.3%	1.00	1.00

Abbreviation: *OR* odds ratio

Model 1 was an unadjusted model

Model 2 was adjusted for gender, age, education level, occupation, marital status, and BMI

^*^ *p* < 0.05;^**^ *p* < 0.01

## Discussion

To our knowledge, this is the first study conducted in an Asian city to examine the association between perceived activity-supportive environmental factors and a specific transport-related sedentary behavior, motorcycle use. Our findings have substantial implications for potential environmental and policy interventions in Taiwan for lowering motorcycle use. They suggest that improving perceptions of access to destinations and public transportation, presence of sidewalks, and safety from crimes at night would be effective in lowering motorcycle use among the urban adult population of Taiwan.

Our results are consistent with studies [17–19] in which certain perceived environmental factors were associated with a specific form of sedentary behavior (i.e., motorcycle use) in urban areas of several developed countries, such as the United States, Australia, and Germany. The analyses revealed that four perceived environmental factors—good access to destinations, public transportation, presence of sidewalks, and safety from crimes at night—were associated with less likelihood of motorcycle use. After adjustment for the sociodemographic variables, the same four environmental factors were still significantly associated with motorcycle use. Moreover, a positive relationship has been reported between walking and cycling and these perceived neighborhood attributes [20–22, 30]. Our results suggest that positive perceptions of the neighborhood environment are associated with not only increased amounts of physical activity, as previously evidenced, but also decreased sedentary time during transport. In addition, residents who perceived a safe neighborhood environment with the presence of sidewalks, good access to local destinations, and public transport might be encouraged to walk or cycle in their neighborhood rather than use motorcycles. In other words, if residents who perceived a safe neighborhood environment with good public transport infrastructure, they might be easier to select other transport mode, such as walking and cycling, instead of motorcycle use [31, 32]. The health risks of sedentary transport in various counties have been reported [8–10]. The findings from this study are unique and substantially contribute to the literature on health risks associated with sedentary transport (i.e., motorcycle use) in a non-Western country. Further research using longitudinal designs are necessary to validate these results.

This study has some limitations. First, causality could not be determined primarily because of the cross-sectional design of this research. Second, the main measurements were self-reported and could be biased [33]. Third, a potential confounder—the self-selection of neighborhoods—was not examined in this study. For instance, those who prefer engaging in physical activities might choose a relatively activity-supportive neighborhood. The neighborhood selection depends on the attributes of the individual rather any association with motorcycle use. Fourth, one-third of the respondents who did not own a household motorcycle did not report riding a motorcycle in the past 7 days, which is likely related with access to a motorcycle. However, because owning a motorcycle is relatively inexpensive and parking is convenient and possibly free, Taiwan has a high motorcycle ownership rate [7]. Therefore, we reasonably presumed that motorcycle ownership might not necessarily be related to the perceptions of activity-supportive environment. Furthermore, although sole car users might be correlated with similar environmental factors [34, 35], we selected non-motorcycle users as a reference group limited by our sample size. Following previous studies [8–10], we presumed that the ORs for this group would be higher than those of our findings. Thus, we suggest that further large-scale research is necessary and encouraged. Finally, this study had a limited representative population sample because it relied on a telephonic survey. Hence, sections of the population without a household telephone (approximately 5.3% in 2013) were not surveyed in this study [36]. Moreover, compared with the national average levels of education and BMI [37–39], the respondents in the present study were more educated and had a higher prevalence of being overweight. Thus, the results of this study cannot be generalized to the entire population of Taiwan.

## Conclusion

Urban Taiwanese adults who perceived their neighborhood environment to have good access to destinations and to public transportation, presence of sidewalks, and safety from crimes at night are less likely to use motorcycles. These perceived neighborhood factors, which have previously been associated with facilitating active transportation, such as walking and cycling, also discourage sedentary transport, such as motorcycling.

## References

[1] Dunstan DW, Howard B, Healy GN, Owen N (2012). Too much sitting–a health hazard. Diabetes Res Clin Pract.

[2] Owen N, Healy GN, Matthews CE, Dunstan DW (2010). Too much sitting: the population health science of sedentary behavior. Exerc Sport Sci Rev.

[3] Owen N, Salmon J, Koohsari MJ, Turrell G, Giles-Corti B (2014). Sedentary behaviour and health: mapping environmental and social contexts to underpin chronic disease prevention. Br J Sports Med.

[4] Taiwan Ministry of Transportation and Communication (2014). 2013 daily transportation report.

[5] Australian Bureau of Statistics (2009). Environmental issues: waste management and transport use.

[6] McKenzie B, Rapino M (2011). Commuting in the US: 2009 American community survey reports.

[7] Taiwan Ministry of Transportation and Communication (2010). Transportation policy white paper.

[8] McCormack GR, Virk JS (2014). Driving towards obesity: a systematized literature review on the association between motor vehicle travel time and distance and weight status in adults. Prev Med.

[9] Sugiyama T, Ding D, Owen N (2013). Commuting by car: weight gain among physically active adults. Am J Prev Med.

[10] Warren TY, Barry V, Hooker SP, Sui X, Church TS, Blair SN (2010). Sedentary behaviors increase risk of cardiovascular disease mortality in men. Med Sci Sports Exerc.

[11] Foster S, Pereira G, Christian H, Knuiman M, Bull F, Giles-Corti B (2015). Neighborhood correlates of sitting time for Australian adults in new suburbs results from RESIDE. Environ Behav.

[12] Koohsari MJ, Sugiyama T, Sahlqvist S, Mavoa S, Hadgraft N, Owen N (2015). Neighborhood environmental attributes and adults’ sedentary behaviors: review and research agenda. Prev Med.

[13] Cervero R, Murakami J (2010). Effects of built environments on vehicle miles traveled: evidence from 370 US urbanized areas. Environ Plan A.

[14] Frank LD, Saelens BE, Powell KE, Chapman JE (2007). Stepping towards causation: do built environments or neighborhood and travel preferences explain physical activity, driving, and obesity?. Soc Sci Med.

[15] Koohsari MJ, Sugiyama T, Kaczynski AT, Owen N (2014). Associations of leisure-time sitting in cars with neighborhood walkability. J Phys Act Health.

[16] Sugiyama T, Cerin E, Owen N (2014). Perceived neighbourhood environmental attributes associated with adults′ recreational walking: IPEN adult study in 12 countries. Health Place.

[17] Van Dyck D, Cerin E, Conway TL (2012). Associations between perceived neighborhood environmental attributes and adults’ sedentary behavior: findings from the USA, Australia and Belgium. Soc Sci Med.

[18] Wallmann-Sperlich B, Bucksch J, Hansen S, Schantz P, Froboese I (2013). Sitting time in Germany: an analysis of socio-demographic and environmental correlates. BMC Public Health.

[19] Shibata A, Oka K, Sugiyama T (2015). Perceived neighbourhood environmental attributes and prospective changes in TV viewing time among older Australian adults. Int J Behav Nutr Phys Act.

[20] Sugiyama T, Shibata A, Koohsari MJ (2015). Neighborhood environmental attributes and adults’ maintenance of regular walking. Med Sci Sports Exerc.

[21] Kerr J, Emond JA, Badland H (2015). Perceived neighborhood environmental attributes associated with walking and cycling for transport among adult residents of 17 cities in 12 countries: the IPEN study. Environ Health Perspect.

[22] Liao Y, Wang I, Hsu HH, Chang SH (2015). Perceived environmental and personal factors associated with walking and cycling for transportation in Taiwanese adults. Int J Environ Res Public Health.

[23] Thomas R, Purdon S (1994). Telephone methods for social surveys. Social research update.

[24] Bergman P, Grjibovski AM, Hagströmer M, Sallis JF, Sjöström M (2009). The association between health enhancing physical activity and neighbourhood environment among Swedish adults—a population-based cross-sectional study. Int J Behav Nutr Phys Act.

[25] Inoue S, Murase N, Shimomitsu T (2009). Association of physical activity and neighborhood environment among Japanese adults. Prev Med.

[26] Santos R, Silva P, Santos P, Ribeiro JC, Mota J (2008). Physical activity and perceived environmental attributes in a sample of Portuguese adults: results from the Azorean physical activity and health study. Prev Med.

[27] The International Physical Activity Prevalence Study (2016). Environmental Module.

[28] Liao Y, Sugiyama T, Shibata A, et al. Associations of perceived and objectively-measured neighborhood environmental attributes with leisure-time sitting for transport. J Phys Act Health. 2016:1–18. 10.1123/jpah.2016-0073 27618495

[29] Health Promotion Administration, Ministry of Health and Welfare Taiwan. Body mass index test. http://health99.hpa.gov.tw/OnlinkHealth/Onlink_BMI.aspx ; 2012 Accessed 28, December, 2016.

[30] Brownson RC, Hoehner CM, Day K, Forsyth A, Sallis JF (2009). Measuring the built environment for physical activity: state of the science. Am J Prev Med.

[31] Salmon J, Owen N, Crawford D, Bauman A, Sallis JF (2003). Physical activity and sedentary behavior: a population-based study of barriers, enjoyment, and preference. Health Psychol.

[32] Evenson KR, Birnbaum AS, Bedimo-Rung AL (2006). Girls’ perception of physical environmental factors and transportation: reliability and association with physical activity and active transport to school. Int J Behav Nutr Phys Act.

[33] Liou YM (2006). The manual of the short-telephone version of international physical activity questionnaires by a computer assisted telephone interviewing (Cati) system.

[34] Cullinane S, Cullinane K (2003). Car dependence in a public transport dominated city: evidence from Hong Kong. Transportation Research Part D-Transport and Environment.

[35] Giuliano G, Dargay J (2006). Car ownership, travel and land use: a comparison of the US and great Britain. Transportation Research Part a-Policy and Practice.

[36] Taiwan Directorate-General of Budget Accounting and Statistics (2013). Report on the survey of family income and expendture.

[37] Taiwan Ministry of Health and Welfare (2013). Nutrition and health survey in Taiwan in 2013.

[38] National statistics, Taiwan (2013). Motor vehicle administration statistics.

[39] Taiwan Ministry of the Interior Department of Statistics (2013). Population statistics in 2013.

